# Comment on ‘*MicroRNA-214* suppresses growth, migration and invasion through a novel target, high mobility group AT-hook 1, in human cervical and colorectal cancer cells’

**DOI:** 10.1038/bjc.2016.409

**Published:** 2016-12-20

**Authors:** Ion Cristóbal, Blanca Torrejón, Juan Madoz-Gúrpide, Federico Rojo, Jesús García-Foncillas

**Affiliations:** 1 Translational Oncology Division, Oncohealth Institute, Health Research Institute FJD-UAM, University Hospital ‘Fundacion Jimenez Diaz’, Madrid, Spain; 2 Department of Pathology, IIS ‘Fundación Jiménez Diaz’, Madrid E-28040, Spain

**Sir,**

We have read with great interest the recently published manuscript by Chandrasekaran *et al* (2016) about the significance of miR-214 in colorectal cancer (CRC). The authors identified high mobility group AT-hook 1 (HMGA1) as a novel direct target of miR-214 and found that low miR-214 levels correlates with high HMGA1 expression in CRC tissues. Moreover, they showed that ectopic miR-214 expression or HMGA silencing led to reduced *in vitro* proliferation, migration and invasion abilities in CRC cells. Although these findings are interesting and of potential importance, there are several limitations in this study that the reader should be taken into account to interpret the conclusions.

Thus, the authors demonstrated by luciferase assays that HMGA1 is a direct miR.-214 target. However, they failed to show by western blot a proper HMGA1 decrease after miR-214 overexpression in the CRC cell lines SW480 and SW620, only obtaining 11% and 7% HMGA1 reduction, respectively. This observation is probably because HMGA1 has been reported to be a target of several microRNAs in human cancer (Kaddar *et al*, 2009; Wei *et al*, 2011; D'Angelo *et al*, 2012; Lau *et al*, 2012; Lin *et al*, 2013; Schubert *et al*, 2013; Xu *et al*, 2014; Zhang *et al*, 2016; Zhou *et al*, 2016), some of those deregulated in CRC, which could be contributing to modulate HMGA1 expression.

Furthermore, the experimental results provided do not permit to claim that the observed antitumor effects after ectopic miR-214 expression are due to its role as HMGA1 regulator. Of note, the similar results described by Chandrasekaran *et al* (2016) in their manuscript could be explained by the fact that HMGA1 positively regulates the Wnt/*β*-catenin pathway by increasing the *β*-catenin-TCF4 complex formation (Xing et al, 2014). Interestingly, miR-214 has also been reported to modulate the Wnt/*β*-catenin pathway targeting *β*-catenin directly or indirectly through EZH2 (Xia *et al*, 2012). To demonstrate that miR-214 exerts its effects through a negative HMGA1 regulation it would be desirable to ectopic express miR-214 in CRC cells ectopically expressing HMGA1 and after its silencing.

Another relevant issue is that there are contradictory data in the literature regarding the HMGA1 status in CRC patients. The work by Liang *et al* (2013) reported that HMGA1 levels are reduced in CRC samples compared with adjacent normal mucosa. However, other studies highlight that HMGA1 contributes to CRC carcinogenesis and serves as a marker of poor prognosis and CRC progression to metastatic disease (Takahashi *et al*, 2013; Xing *et al*, 2014; Williams *et al*, 2015). Unfortunately, Chandrasekaran *et al* (2016) only analyzed HMGA1 in CRC samples at the mRNA level. Considering the number of microRNAs involved in HMGA1 regulation and that only a perfect match between the microRNA and its target leads to mRNA degradation, one would not expect a good correlation between HMGA1 mRNA and protein levels. This mRNA-protein correlation for HMGA1 was not analyzed in their work, and then only the evaluation of HMGA1 at the protein levels could have helped to clarify its status in CRC patients. Additionally, it woculd also be of interest to know the clinical and molecular characteristics of the patient cohort included in this study.

In conclusion, HMGA1 is under a complex regulation involving several different microRNAs, and miR-214 alone does not seem to be able to exert enough changes in HMGA1 expression to sustain that its biological significance is due to this effect. Further studies are needed to fully clarify the role of miR-214 and the HMGA1 status in CRC, and evaluate their potential therapeutic value as novel molecular targets in this disease.
